# Stable complete methane oxidation over palladium based zeolite catalysts

**DOI:** 10.1038/s41467-018-04748-x

**Published:** 2018-06-29

**Authors:** Andrey W. Petrov, Davide Ferri, Frank Krumeich, Maarten Nachtegaal, Jeroen A. van Bokhoven, Oliver Kröcher

**Affiliations:** 1 Paul Scherrer Institut (PSI), CH-5232 Villigen, Switzerland; 2 ETH Zurich, Institute for Chemical and Bioengineering, CH-8093 Zurich, Switzerland; 3 École polytechnique fédérale de Lausanne (EPFL), Institute of Chemical Sciences and Engineering, CH-1015 Lausanne, Switzerland

## Abstract

Increasing the use of natural gas engines is an important step to reduce the carbon footprint of mobility and power generation sectors. To avoid emissions of unburnt methane and the associated severe greenhouse effect of lean-burn engines, the stability of methane oxidation catalysts against steam-induced sintering at low temperatures (<500 °C) needs to be improved. Here we demonstrate how the combination of catalyst development and improved process control yields a highly efficient solution for complete methane oxidation. We design a material based on palladium and hierarchical zeolite with fully sodium-exchanged acid sites, which improves the support stability and prevents steam-induced palladium sintering under reaction conditions by confining the metal within the zeolite. Repeated short reducing pulses enable the use of a highly active transient state of the catalyst, which in combination with its high stability provides excellent performance without deactivation for over 90 h in the presence of steam.

## Abstract (web summary)

Palladium supported on zeolite is a highly active catalyst for complete methane oxidation, but its stability needs to be improved. Here, the authors design a highly active catalyst resistant to steam-induced sintering under reaction conditions by alleviating the high mobility of palladium nanoparticles and zeolite degradation.

## Introduction

Increasingly stringent emission regulations in the automotive sector force transition to cleaner fuels and more efficient combustion processes. Lean-burn natural gas vehicles are a promising mid-term alternative to gasoline and diesel vehicles due to the higher mass energy density of methane and lower carbon dioxide and NO_x_ emissions^1–5^. However, lean-burn operation of a natural gas engine results in incomplete combustion of methane, the major component of natural gas. The 20 times larger global warming potential than carbon dioxide makes the control of methane emissions compulsory^6–9^. For this purpose, catalytic exhaust gas aftertreatment is used, which is currently based on conventional materials like Pd/Al_2_O_3_ and its modification by ceria, Pd/SiO_2_, Pd/ZrO_2_, and Pd/SnO_2_^10–12^. However, such systems have invariably poor hydrothermal stability, especially under realistic steady state operation at low temperature where steam-induced palladium sintering represents the major reason for deactivation^13–15^. Yet very few papers address this issue, preferring to focus on achieving the lowest possible light-off temperature in dry conditions and neglecting the problem of the steam-induced sintering^1,16,17^, although practical applications always involve the presence of large amounts of steam. Developing materials, which remain highly active during exposure to severe conditions with high steam concentration is essential to implement the use of natural gas-powered vehicles and power generation systems^15,18,19^.

Palladium supported on zeolite is a highly active material for complete methane oxidation^2,15,20,21^. Zeolites offer several advantages compared to metal oxide supports, the most fascinating one being the possibility to constrain small metal particles to protect them from sintering^22,23^, which is otherwise achieved upon laborious chemical treatment^1,10,11^. This opportunity has not been exploited so far for this reaction using zeolites. Low-silica zeolites containing sodium were found to be active but showed poor stability under reaction conditions in the presence of steam^20,21^. High silicon to aluminum (Si/Al) ratios in Pd/ZSM-5 and Pd/beta are beneficial for the activity and stability due to higher hydrophobicity and stability of the zeolite support^24–27^. However, achieving high dispersion of palladium within high silica zeolites is challenging due to their high hydrophobicity and small ion-exchange capacity, which do not allow incorporation of palladium into the zeolite pores. On the contrary, high dispersion even at high loadings of the active phase can be achieved at low Si/Al ratios^26^. While being advantageous for the synthesis in oxidizing conditions, the increased acidity of the zeolite could enhance the mobility of palladium over the support under reaction conditions, which include methane and large amounts of steam in the feed, eventually resulting in particle growth^13^. In addition, dealumination degrades the zeolite crystallinity and leads to the formation of acidic extra-framework aluminum species, which further promote palladium sintering^13,28^. Provided that the active palladium phase can be kept stable at high dispersion, the use of low-silica zeolite thus provides a unique opportunity to achieve high loading and high dispersion of the metal.

In this work, we design a highly active catalyst resistant to steam-induced sintering under reaction conditions by alleviating the origins of catalyst deactivation: high mobility of palladium nanoparticles and zeolite degradation. Both issues are solved by the synthetic approach based on the complete removal of the acid sites of the zeolite by post-exchange with sodium with simultaneous confinement of palladium nanoparticles within the zeolite. Additionally, with the help of operando X-ray absorption spectroscopy (XAS) revealing the beneficial redox properties of the catalyst, i.e., rapid palladium reduction and slow reoxidation, a new operation protocol is proposed, which maximizes the presence of a highly active transient state and provides stable conversion of methane for over 90 h in hydrothermal conditions with a negligible increase in methane emission.

## Results and Discussion

### Control of acidity by titration with sodium

**Fig. 1:**
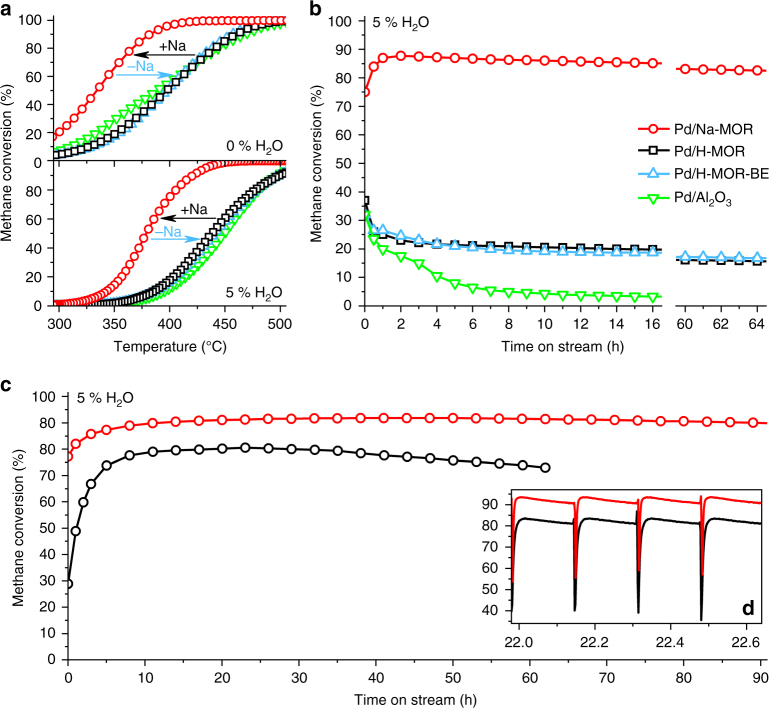
Catalytic activity of Pd/Al_2_O_3_ and various Pd/mordenite catalysts. **a** Light-off curves, **b** 65 h stability test without regeneration, and **c** stability test with continuous short pulse regeneration (averaged values including methane slip during regeneration). Conditions: 1 vol% CH_4_, 4 vol% O_2_, 0 or 5 vol% H_2_O, bal. N_2_; gas hourly space velocity (GHSV) = 70,000 h^−1^; *T* = 415 °C for stability tests; lean operation: 10 min; regeneration (short rich operation): 3 s; **d** magnification of four cycles with regeneration over Pd/H-MOR and Pd/Na-MOR. Prior to the tests, the catalysts were degreened at 550 °C for 30 min in 1 vol% CH_4_, 4 vol% O_2_, 95 vol% N_2_

The Pd/H-MOR catalyst was prepared by ion-exchange of H-mordenite with tetraammine palladium nitrate followed by calcination in air at 500 °C. Fully sodium-exchanged Pd/Na-MOR was obtained by titrating the residual acid sites of Pd/H-MOR with a 0.01 M solution of sodium bicarbonate. Pd/H-MOR and a reference 1 wt% Pd/Al_2_O_3_ catalyst prepared by wet impregnation showed similar light-offs in lean-burn methane oxidation, both providing 50% methane conversion (*T*_50_, Fig. 1a, Table 1) at 400 °C and 435 °C, in the absence and presence of water vapor, respectively. The corresponding *T*_50_ values of Pd/Na-MOR shifted considerably to 340 and 375 °C, and its intrinsic activity was identical to the most active palladium-based catalyst reported in literature (Supplementary Fig. 1)^1^. To cross-check the possible influence of acidity removal on deactivation of the system and exclude any other factor, such as change in the pore structure and crystallinity of the support material, Pd/Na-MOR was back-exchanged with ammonium nitrate and calcined to obtain the acidic Pd/H-MOR-BE. The *T*_50_ values of this catalyst (400/440 °C) were close to the *T*_50_ values of Pd/H-MOR.

**Table 1:** Physico-chemical properties of Pd/MOR catalysts

Sample	Si/Al ratio^a^	Na^a^, wt%	Pd^a^, wt%	Surface area^b^, m^2^/g (calcined/spent 16 h)	Crystallinity^c^, % (spent 16 h)	Strong acid sites^d^, mmol/g
Pd/H-MOR	17.2	0.06	1.01	418/414	93	0.62
Pd/Na-MOR	17.2	1.43	0.99	400/402	93	0.10
Pd/H-MOR-BE	17.2	0.01	0.98	416/415	91	0.54

^a^ From inductively coupled plasma optical emission spectroscopy (ICP-OES)

^b^ From argon physisorption using Brunauer–Emmett–Teller (BET) method

^c^ Calculated from the diffraction peak at 2*θ* = 22.4°, the corresponding calcined materials were taken as 100%

^d^ Integrated value from ammonia desorption peak at 250–550 °C (Supplementary Fig. 5)

Despite the clear water poisoning effect over all catalysts (Fig. 1a), the enhanced activity of Pd/Na-MOR was accompanied by a significant increase in stability (Fig. 1b). The acidic catalysts, Pd/H-MOR and Pd/H-MOR-BE, as well as Pd/Al_2_O_3_, exhibited rapid deactivation within the first 4 h on stream, in agreement with observations by others^14,15,20^. On the contrary, methane conversion over Pd/Na-MOR improved and the initial 35% difference in conversion increased to 65% after 4 h. Negligible variations in crystallinity (X-ray diffraction, XRD; Supplementary Fig. 2, Supplementary Table 1), pore structure and surface area (argon physisorption, Supplementary Fig. 3, Supplementary Table 2), and aluminum coordination (^27^Al MAS NMR, Supplementary Fig. 4) in the three catalysts suggest that addition and removal of sodium to Pd/H-MOR did not affect the structure of the zeolite, but changed its acidity. This was also inferred from ammonia desorption profiles (Table 1, Supplementary Fig. 5, Supplementary Table 3) and the vibrational fingerprints of ammonia adsorption/desorption in infrared spectroscopy (Supplementary Fig. 6). The similarity between Pd/H-MOR and Pd/H-MOR-BE and the superior performance of fully exchanged Pd/Na-MOR suggest that the acidity of the zeolite controls the catalytic behavior of the system. This was further confirmed by the extensive study of the effect of sodium loading on the activity of Pd/MOR (Supplementary Table 4), which showed that the optimal amount of sodium corresponded to the fully exchanged acid sites of the zeolite. Addition of insufficient amounts of sodium did not significantly improve the performance of Pd/MOR, whereas an excess of sodium caused rapid deactivation of the catalyst. Despite the possible influence of sodium on kinetics and on the reaction mechanism^29^, no significant difference in reaction orders and activation energy was observed between Pd/H-MOR and Pd/Na-MOR (Table 2). Hence, some other factor is responsible for the improved performance of Pd/Na-MOR.

**Table 2:** Kinetic analysis of Pd/H-MOR and Pd/Na-MOR

Sample	Reaction orders (CH_4_/O_2_/H_2_O)	*E*_act_, kJ/mol (0/5 vol% H_2_O)	*T*_50_, °C (0/5 vol% H_2_O)	TOF^a^, s^−1^ (275 °C, 0% H_2_O)	TOF^a^, s^−1^ (340 °C, 5% H_2_O)
Pd/H-MOR	0.9/0.1/−1.0	84/155	395/435	0.003	0.002
Pd/Na-MOR	1.0/0.1/−1.1	75/158	335/375	0.013	0.014

^a^ Turnover frequency (TOF) calculated per surface atom of palladium

**Fig. 2:**
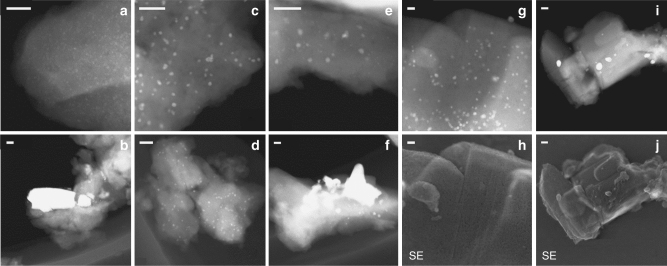
Representative electron microscopy images of calcined and spent catalysts. **a**, **b** Pd/H-MOR, **c**, **d** Pd/Na-MOR, **e**, **f** Pd/H-MOR-BE. Spent catalysts were aged in 1 vol% CH_4_, 4 vol% O_2_, 5 vol% H_2_O, N_2_ bal. at 415 °C for 16 h. STEM and secondary electron (SE) images of **g**, **h** Pd/Na-MOR and **i**, **j** Pd/H-MOR after 90 h and 65 h on stream, respectively, continuous with short pulse regeneration. Scale bar: 20 nm (**a**–**h**) and 50 nm (**i**, **j**)

The scanning transmission electron microscopy (STEM) images of Pd/H-MOR (Fig. 2a) exhibited homogeneously distributed palladium nanoparticles with a mean diameter of 1.3 nm (Supplementary Fig. 7). Removal of acidity by sodium exchange (Fig. 2c) resulted in a moderate aggregation of palladium and the mean diameter of the metal particles of Pd/Na-MOR increased to 3.0 nm. The decrease of the mean particle size to 2.4 nm after removal of sodium (Pd/H-MOR-BE) evidenced the partial redispersion of palladium, accompanied by restoration of the support acidity (Supplementary Fig. 5). Figure 2e shows two distinct particle sizes in Pd/H-MOR-BE: the smaller particles (1–1.5 nm) similar to those in the parent Pd/H-MOR and the larger ones (2–4 nm) like in Pd/Na-MOR. This aggregation–redispersion phenomenon was also confirmed by analysis of the ex situ Pd K-edge extended X-ray absorption fine structure (EXAFS) spectra (Supplementary Fig. 8) and agrees with the previous observations on Pd/H-zeolite and Pd/Na-zeolite^30^, where palladium tended to stay more aggregated on the sodium-exchanged zeolite. Such behavior suggests that palladium can actively interact with the acid sites of the zeolite, which can promote mobility of the metal over the support in the presence of water vapor under reaction conditions^13^. STEM micrographs of the catalysts exposed to reaction conditions for 16 h revealed sintering of the active phase in Pd/H-MOR and Pd/H-MOR-BE (Fig. 1b,f) with highly inhomogeneous particle size ranging from 3 to 50 nm (Supplementary Fig. 7) and the average nanoparticle size was 14 nm and 11 nm, respectively. On the contrary, no significant change in palladium dispersion occurred in Pd/Na-MOR (an average of 3.0 nm for the calcined material, and 4.4 nm and 4.6 nm for samples spent for 16 h and 65 h, respectively).

The micrographs of Pd/Na-MOR (Fig. 2c, d) exhibited a large fraction of palladium nanoparticles within the dark areas of the zeolite crystallites, which are typically recognized as mesopores^31^. The location of these particles was confirmed to be the zeolite interior by STEM with simultaneous secondary electron detection (Fig. 2g, h), whereas only a few particles were detected on the external surface of the zeolite. The native H-mordenite with a Si/Al ratio of 17 used in this study was dealuminated by the supplier and it is well-known that dealumination of zeolites produces mesopores^32–36^. Exchange of sodium likely caused aggregation of palladium within such cavities of the support, trapping the active phase in a well-dispersed form within the zeolite^37^. The acid sites, which caused internal exchange and mobility of palladium in the presence of water vapor, were removed in Pd/Na-MOR, and this, thus ensured that palladium remained confined within the zeolite.

### Redox properties and transient state

**Fig. 3:**
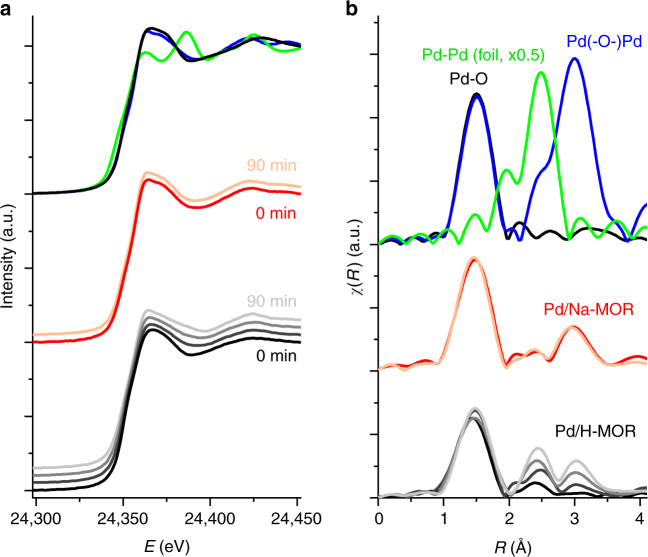
In situ X-ray absorption spectroscopy of catalyst aging. **a** X-ray absorption near-edge structure (XANES) spectra and **b** the corresponding Fourier transforms of in situ Pd K-edge extended X-ray absorption fine structure (EXAFS) spectra (non-phase shift corrected) of the catalysts after pretreatment (10 min in 1 vol% CH_4_, 4 vol% O_2_, bal. N_2_, GHSV = 350,000 h^−1^ at 410 °C) and after 15, 30, and 90 min aging in a feed of 1 vol% CH_4_, 4 vol% O_2_, 5 vol% H_2_O, bal. N_2_ at 410 °C: Pd/H-MOR (black to light gray); Pd/Na-MOR (red to pink); bulk PdO (blue); calcined Pd/H-MOR (black); and palladium foil (green). The spectra and Fourier transforms are offset for clarity

The increase in the intensities and narrowing of the peaks of palladium oxide reflections in the XRD patterns of the spent Pd/H-MOR and Pd/H-MOR-BE catalysts compared to the calcined ones (Supplementary Fig. 2) support the conclusions about the particle size drawn from STEM. Additionally, both palladium oxide and metallic palladium were detected in the spent acidic catalysts (Pd/H-MOR and Pd/H-MOR-BE), whereas only palladium oxide was present in fresh and spent Pd/Na-MOR. To further study the observed difference in the oxidation state of palladium and elucidate the possible influence of sodium, Pd/H-MOR and Pd/Na-MOR were exposed to the reaction conditions at 400 °C for 90 min while measuring in situ Pd K-edge EXAFS. The Fourier transforms (FT) of the spectra obtained at selected time intervals (Fig. 3, Table 3) show a gradual increase in the Pd–Pd (peak in FT at 2.5 Å, non-phase shift corrected) and Pd(–O–)Pd (peak in FT at 3.0 Å, non-phase shift corrected) coordination shells of Pd/H-MOR over time^26^. The fractions of oxidized and reduced palladium were determined from the fractional coordination numbers. Assuming that the coordination number of Pd–O (CN_Pd–O_) is 4 (CN fitting error here and below is ±0.6), the value of CN_Pd–O_ ≈ 3 in Pd/H-MOR corresponded to about 75% of oxidic palladium. The coordination number of fully coordinated metallic palladium (CN_Pd–Pd_) at this reduction level would be 3 and therefore the value of 2.8 in Pd/H-MOR aged for 90 min indicates that reduced palladium was organized in large particles with an average coordination number of about 11. In contrast, no significant difference in the state of palladium was observed in Pd/Na-MOR after 90 min suggesting that it always remained fully oxidized (CN_Pd–O_ ≈ 4).

**Table 3:** Coordination numbers obtained from fitting the EXAFS spectra

Experiment	CN_Pd-O_^a^	CN_Pd-Pd_	CN_Pd(-O-)Pd_^b^	Fract. CN_Pd-Pd_^c^	Fract. CN_Pd(-O-)Pd_^c^
Ex situ, 20 °C					
PdO	4	–	12	–	12
Pd foil	–	12		12	–
Pd/H-MOR	4	–	–	–	–
Pd/Na-MOR	4	–	6	–	6
Pd/H-MOR-BE	4	–	3.3	–	3.3
Aging at 400 °C (Fig. 3) Pd/H-MOR					
0 min	2.9	0.5	1.0	1.8	1.4
15 min	2.9	1.6	2.7	5.8	3.7
30 min	2.9	1.9	4.2	6.9	5.8
90 min	3	2.8	7.0	11.2	9.3
Pd/Na-MOR					
0 min	3.9	–	6.0	–	6.2
90 min	3.9	–	6.6	–	6.8
Redox at 350 °C (Fig. 4) Pd/H-MOR					
1 s	2.4	4.6	5.2	11.5	8.7
90 s	–	11.3	–	11.3	–
210 s	–	11.3	–	11.3	–
280 s	1.4	7.0	3.9	10.8	11.1
550 s	2.3	4.9	6.3	11.5	11.0
Pd/Na-MOR					
1 s	3.9	–	6.7	–	6.9
90 s	–	10.2	–	10.2	–
210 s	–	10.4	–	10.4	–
280 s	2.4	3.6	2.3	9.0	3.8
550 s	3.6	1.2	6.9	12.0	7.7

^a^ The absolute error value of the fitted coordination numbers is in the order of ±0.6

^b^ The Pd(−O−)Pd shell is a sum of two scattering paths with the maximum coordination numbers of 4 and 8

^c^ The fractional coordination numbers are obtained from normalization by the fraction of reduced/oxidized palladium from CN_Pd−O_

**Fig. 4:**
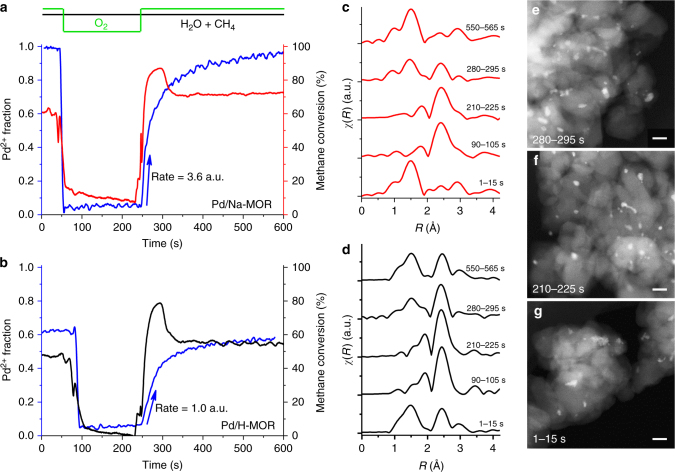
Transient *operando* EXAFS experiment with oxygen cut-off. **a**, **b** Fraction of oxidized palladium in Pd/Na-MOR and Pd/H-MOR and corresponding catalytic activity in 1 vol% CH_4_, 4 vol% O_2_, 5 vol% H_2_O, bal. N_2_, GHSV = 350,000 h^−1^ at 350 °C. Oxygen was removed from the feed to perform reduction of palladium by methane and then added for subsequent reoxidation in reaction conditions. **c**, **d** Fourier transforms of in situ Pd K-edge EXAFS spectra (non-phase shift corrected) of Pd/Na-MOR and Pd/H-MOR upon averaging 15 spectra at 1, 90, 210, 280, and 550 s of the experiment. Corresponding coordination numbers are given in Table 2. Initial rates were obtained from fitting the linear function over the first 10 points during reoxidation. **e**, **f**, **g** STEM images of the quenched Pd/H-MOR at 1, 210, and 280 s. Scale bar: 20 nm

Subsequent to the steady state operation (90 min at 400 °C), we assessed the redox properties of palladium in the absence and presence of sodium in a transient time-resolved XAS experiment (Fig. 4). Oxygen was removed from the feed to reduce the catalyst by methane and then added again to allow palladium reoxidation under reaction conditions. The results of the XANES linear combination fit, illustrated in Fig. 4a, show that after removal of oxygen the initially fully oxidized aged Pd/Na-MOR was completely reduced within the first few seconds. In the reduction period, both catalysts continued to convert methane to carbon dioxide and hydrogen by steam reforming. When oxygen was re-introduced, the catalyst slowly reoxidized and contained 95% of Pd^2+^ after 5 min of reoxidation. Similarly, the complete reduction of initially partly reduced aged Pd/H-MOR (62% of Pd^2+^) occurred upon the removal of oxygen, followed by the slow reoxidation under reaction conditions. After 5 min, Pd/H-MOR had a slightly lower fraction of Pd^2+^ (58%) than before reduction. Despite the similar behavior of Pd/H-MOR and Pd/Na-MOR during reduction and reoxidation, the steady state fraction of Pd^2+^ under reaction conditions was significantly higher in Pd/Na-MOR. We attribute this behavior to about four times faster reoxidation rate of Pd^0^ to Pd^2+^ in the presence of sodium (the initial rates are shown in Fig. 4), considering that smaller metal nanoparticles with larger specific surface area are generally more reactive towards oxygen^38,39^, and that the palladium particle size was smaller in Pd/Na-MOR as inferred from the CN_Pd–Pd_ of the fully reduced catalysts (90 and 210 s; Table 3, Supplementary Figs. 9 and 10).

After oxygen was re-introduced, methane conversion over Pd/H-MOR and Pd/Na-MOR increased transiently to 78% and 86%, respectively, and then stabilized at a higher steady state activity level than before reduction. The increased activity upon reoxidation of reduced palladium can be attributed either to the formation of a highly dispersed PdO phase (redispersion), to the increased surface roughness of freshly reoxidized PdO, or to the presence of the interface between the reduced and oxidized palladium^40^. The coordination numbers of palladium in Pd/Na-MOR (Table 3) showed that under reducing conditions (210 s) palladium existed as large reduced particles, which quickly partially reoxidized as soon as oxygen was added to the feed (280 s). Peculiarly, the Pd(–O–)Pd shell of palladium in Pd/Na-MOR was almost undetectable in this transient state, which implies that palladium oxide existed as very small entities. STEM analysis (Fig. 4e-g, Supplementary Figs. 11 and 12) confirmed that no redispersion of palladium occurred and the particles exhibited irregular morphology both before and after the reducing pulse, suggesting that the difference in the particle shape is unlikely the reason for the high transient activity. According to some previous work, the PdO–Pd interface is unlikely to provide enhanced methane oxidation activity due to the strongly chemisorbed oxide layer on metallic palladium^41,42^, and therefore the high activity in the transient state may occur due to the increased amount of oxygen vacancies^43,44^ in freshly reoxidized palladium caused by volume change and surface roughening^40,45^. The extinction of the transient state was followed by a slower reoxidation rate of palladium, suggesting that the surface of palladium fully reoxidized and further oxidation was suppressed by the oxygen diffusion to the bulk of palladium particles. The system reached a new steady state, in which Pd/H-MOR and Pd/Na-MOR showed 8% and 15% higher methane conversion than before reduction (1 s), respectively. The incomplete reoxidation of Pd/H-MOR operated under the steady state can be explained by the above described phenomenon of fast surface and slow bulk reoxidation of its large particles, which likely produced bulk metal particles coated by an oxide shell^46^. Interestingly, the effect of redox pulsing on Pd/H-MOR with large particles was much more pronounced compared to highly dispersed Pd/Na-MOR, suggesting that the activity of the transient state does not correlate with palladium particle size. Such effect is not surprising, since activity of the catalyst in the active transient state is defined by the surface roughness and the amount of oxygen vacancies in the freshly reoxidized PdO.

### Practical implications

The enhanced conversion in the transient state can be exploited for practical use to prevent the long-term deactivation. While reducing treatments are known to regenerate poisoned catalysts after steady-state operation^47^, the rapid reduction of palladium in the absence of oxygen in the feed demonstrated in this work allows to decrease the reduction time to a few seconds. Thus, instead of the conventional steady state operation, the catalyst can be continuously operated in the highly active transient state provoked by short reducing pulses applied between longer lean periods without a significant increase in methane emissions associated with such reduction. Figure 1c shows the stability of Pd/H-MOR and Pd/Na-MOR during operation with continuous short pulses, wherein oxygen was removed from the feed for 3 s at the intervals of 10 min. This operation mode initially increased the methane oxidation activity over Pd/Na-MOR and allowed to maintain the high conversion level of 90% without a significant change in activity of the catalyst for over 90 h on stream. This high conversion level is maintained, because of the continuous regeneration of the highly active transient state during the reduction/reoxidation cycles identified by operando XAS. Its lifetime in the stationary reactor (Fig. 1c) is longer than in the capillary used for the XAS measurements (Fig. 4a) due to the lower space velocity (70,000 h^−1^) compared to the spectroscopic experiment (350,000 h^−1^). On the contrary, Pd/H-MOR showed deactivation in the stability test, although the repeated short pulses increased the highest conversion of methane to 79% compared to 37% obtained in the conventional mode. Such difference occurred due to the superior sintering resistance of Pd/Na-MOR compared to Pd/H-MOR as evidenced by STEM (Fig. 2g–j). The strategy described above can be readily implemented, since modern engines are designed for switching between lean, stoichiometric, and rich operation modes to optimize fuel consumption and to control exhaust gas aftertreatment devices, which is used in existing technologies for NO_x_ abatement like three-way catalyst (TWC) and lean NO_x_ trap (LNT).

This work shows that palladium can be efficiently stabilized at high dispersion within the mesopores of dealuminated hierarchical mordenite. The mobility of palladium was inhibited by fully exchanging the acid sites of the zeolite with sodium, which kept palladium highly dispersed and prevented steam-induced sintering under reaction conditions. Due to the very rapid reduction and the slow reoxidation of palladium observed by transient operando XAS, it was possible to maintain the catalyst at high transient activity compared to steady state conditions by applying very short reducing pulses of methane between longer lean periods. This sustained the highly active transient state, palladium oxide with increased amount of oxygen vacancies, providing higher steady state activity in methane oxidation and, in combination with the sintering-resistant Pd/Na-MOR, stable methane conversion for over 90 h in hydrothermal conditions.

## Methods

### Materials and synthesis

Commercial dealuminated mordenite was supplied by Zeochem (FM-8/25H, Si/Al = 17, H^+^ form). The zeolite was ion-exchanged with the amount of tetraammine palladium nitrate solution (pH = 7) corresponding to the nominal 1 wt% loading of palladium at room temperature for 24 h. After drying at 120 °C for 6 h, the catalyst was calcined in air at 500 °C for 2 h in a muffle oven. The heating ramp was set to 2 °C min^–1^ to form highly dispersed palladium particles. The resulting material was denoted as Pd/H-MOR.

A batch of Pd/H-MOR (25 g) was dispersed in deionized water (250 ml) and treated with a diluted solution of sodium bicarbonate (0.01 M, pH = 8), which was added dropwise to the slurry (pH = 3.5) within 90 min until it was neutralized (pH = 7). The slurry was then stirred for 30 min, centrifuged to recover the solid, dried at 120 °C for 6 h, and calcined in air at 500 °C for 2 h. The obtained catalyst was labeled as Pd/Na-MOR. The Pd/Na-MOR catalyst (3 g) was then ion-exchanged three times with ammonium nitrate (1 M, 100 ml, 24 h) to back-exchange the sodium followed by drying and calcination procedures as described above. The resulting catalyst was denoted as Pd/H-MOR-BE. Excessively exchanged Pd/Na-MOR-EE was prepared by stirring Pd/H-MOR (2 g) in sodium bicarbonate solution (1 M, 200 ml) for 2 h. The catalyst was then treated identically to Pd/Na-MOR. For the conventional synthesis route, H-MOR (5 g) was exchanged three times with sodium nitrate (1 M, 100 ml), washed with deionized water, dried at 120 °C for 6 h, and calcined at 500 °C for 2 h. The solid was then ion-exchanged with tetraammine palladium nitrate solution (pH = 7 for Pd/NaH-MOR-1 and pH = 5 for Pd/NaH-MOR-2), dried, and calcined as described above. Pd/Al_2_O_3_ was synthesized by wet impregnation. Alumina powder (SASOL Puralox UF 5/230) was stirred for 6 h at 80 °C with a palladium nitrate solution (Alfa Aesar) corresponding to 1 wt% loading of palladium. After the solvent was slowly evacuated, the solid was dried and calcined following the same protocol as for Pd/H-MOR.

### Characterization

The Si/Al ratio, palladium, and sodium contents of the catalysts were determined by inductively coupled plasma optical emission spectrometry (ICP-OES) using a Varian VISTA Pro AX instrument. STEM images were taken with a high-angle annular dark-field detector (HAADF) on an aberration-corrected Hitachi HD-2700 microscope operated at an acceleration voltage of 200 kV. QuickEXAFS data were acquired in transmission mode at the Pd K-edge (24.35 keV) at the SuperXAS beamline of the Swiss Light Source (SLS, Paul Scherrer Institute) at the time resolution of 1 s. The catalytic activity was monitored by on-line mass-spectrometer. The Fourier transformation of the k^2^-weighted EXAFS functions was performed in the range 3–11 Å^−1^. Data reduction was performed using the Athena software package^48^. A Bruker D8 Advance AXS diffractometer with Cu Kα radiation was used to obtain powder XRD patterns in the 2*θ* range from 10° to 60°. Solid-state magic angle spinning nuclear magnetic resonance (MAS NMR) ^27^Al spectra were recorded at 10 kHz (1024 accumulations) on a Bruker Avance 400 MHz spectrometer with ammonium aluminum sulfate dodecahydrate (AlNH_4_(SO_4_)_2_·12H_2_O) as a reference. The textural properties of the support were characterized by argon physisorption at 77 K using a Quantachrome Autosorb 1 instrument after degassing at 300 °C for 24 h to remove water and other adsorbed species. The specific surface area was calculated using the Brunauer–Emmett–Teller (BET) method. Pore size distribution in the range from 0.5 to 50 nm was calculated by fitting the isotherms using the method of non-linear density functional theory (NLDFT) from the instrument software package. Diffuse reflectance Fourier transform infrared (DRIFT) spectra were measured using a Bruker Vertex 70 spectrometer equipped with a Praying Mantis mirror unit and a liquid nitrogen cooled MCT detector. All DRIFT spectra were collected by accumulating 100 scans at 4 cm^−1^ resolution and a scanner velocity of 80 kHz. Prior to acquisition of the background spectrum, the sample was dehydrated at 350 °C for 1 h. NH_3_ adsorption was followed at 100 °C during exposure of the catalyst to 500 ppm of ammonia in nitrogen at the flow rate of 20 ml min^–1^. NH_3_ desorption was monitored while the temperature of the sample was increased from 100 to 300 °C in nitrogen flow.

### Data availability

The datasets generated and analyzed during the current study are archived on the internal servers of the Paul Scherrer Institut and are available from the corresponding authors on reasonable request.

## Electronic supplementary material

Supplementary Information

Peer Review File
