# Supplementary material for: Stable complete methane oxidation over palladium based zeolite catalysts
Source: Nat Commun. 2018 Jun 29;9:2545. doi: 10.1038/s41467-018-04748-x (PMC6026177; doi:10.1038/s41467-018-04748-x)
Supplement: Supplementary file 1 — Supplementary Information [file 41467_2018_4748_MOESM1_ESM.docx]

Stable complete methane oxidation over palladium based zeolite catalysts

by Petrov et al.

Supplementary Information

Supplementary methods

**Catalyst synthesis.** Commercial dealuminated mordenite was supplied by Zeochem (FM-8/25H, Si/Al = 17, H^+^ form). The zeolite was ion-exchanged with the amount of tetraammine palladium nitrate solution (pH = 7) corresponding to the nominal 1 wt. % loading of palladium at room temperature for 24 h. After drying at 120 ^o^C for 6 h, the catalyst was calcined in air at 500 ^o^C for 2 h in a muffle oven. The heating ramp was set to 2 ^o^C per min to form highly dispersed palladium particles. The resulting material was denoted as Pd/H-MOR.

A batch of Pd/H-MOR (25 g) was dispersed in deionized water (250 ml) and treated with a diluted solution of sodium bicarbonate (0.01 M, pH = 8), which was added dropwise to the slurry (pH = 3.5) within 90 min until it was neutralized (pH = 7). The slurry was then stirred for 30 min, centrifuged to recover the solid, dried at 120 ^o^C for 6 h and calcined in air at 500 ^o^C for 2 h. The obtained catalyst was labelled as Pd/Na-MOR. The Pd/Na-MOR catalyst (3 g) was then ion-exchanged three times with ammonium nitrate (1 M, 100 ml, 24 h) to back-exchange the sodium followed by drying and calcination procedures as described above. The resulting catalyst was denoted as Pd/H-MOR-BE. Excessively exchanged Pd/Na-MOR-EE was prepared by stirring Pd/H-MOR (2 g) in sodium bicarbonate solution (1 M, 200 ml) for 2 h. The catalyst was then treated identically to Pd/Na-MOR. For the conventional synthesis route, H-MOR (5 g) was exchanged three times with sodium nitrate (1 M, 100 ml), washed with deionized water, dried at 120 ^o^C for 6 h and calcined at 500 ^o^C for 2 h. The solid was then ion-exchanged with tetraammine palladium nitrate solution (pH = 7 for Pd/NaH-MOR-1 and pH = 5 for Pd/NaH-MOR-2), dried and calcined as described above.

**Catalyst characterization.** The Si/Al ratio, palladium and sodium contents of the catalysts were determined by inductively coupled plasma optical emission spectrometry (ICP-OES) using a Varian VISTA Pro AX instrument. Scanning transmission electron microscopy images (STEM) were taken with a high-angle annular dark-field detector (HAADF) on an aberration-corrected Hitachi HD-2700 microscope operated at an acceleration voltage of 200 kV. Extended X-ray adsorption fine structure (EXAFS) data were acquired in transmission mode at the Pd K-edge (24.35 keV) at the SuperXAS beamline of the Swiss Light Source (SLS, Paul Scherrer Institute) at the time resolution of 1 s. The catalytic activity was monitored by on-line mass-spectrometer. The Fourier transformation of the k^2^-weighted EXAFS functions was performed in the range 3 to 11 Å^-1^. Data reduction was performed using the Athena software package.^1^ A Bruker D8 Advance AXS diffractometer with Cu Kα radiation was used to obtain powder X-ray diffraction (XRD) patterns in the 2θ range from 10^o^ to 60^o^. Solid-state magic angle spinning nuclear magnetic resonance (MAS NMR) ^27^Al spectra were recorded at 10 kHz (1024 accumulations) on a Bruker Avance 400 MHz spectrometer with ammonium aluminum sulfate dodecahydrate (AlNH_4_(SO_4_)_2_·12H_2_O) as a reference. The textural properties of the support were characterized by argon physisorption at 77 K using a Quantachrome Autosorb 1 instrument after degassing at 300 ^o^C for 24 h to remove water and other adsorbed species. The specific surface area was calculated using the Brunauer−Emmett−Teller (BET) method. Pore size distribution in the range from 0.5 to 50 nm was calculated by fitting the isotherms using the method of non-linear density functional theory (NLDFT) from the instrument software package. Diffuse reflectance Fourier transform infrared (DRIFT) spectra were measured using a Bruker Vertex 70 spectrometer equipped with a Praying Mantis mirror unit and a liquid nitrogen cooled MCT detector. All DRIFT spectra were collected by accumulating 100 scans at 4 cm^-1^ resolution and a scanner velocity of 80 kHz. Prior to acquisition of the background spectrum, the sample was dehydrated at 350 ^o^C for 1 h. NH_3_ adsorption was followed at 100 ^o^C during exposure of the catalyst to 500 ppm of ammonia in nitrogen at the flow rate of 20 ml per min. NH_3_ desorption was monitored while the temperature of the sample was increased from 100 to 300 ^o^C in nitrogen flow.

**Catalyst activity evaluation.** The catalytic activity of powder samples (50 mg, 150-200 µm sieve fraction) was evaluated in a dedicated setup consisting of a quartz-glass plug flow reactor (ID = 6 mm), gas flowmeters, heating control and a gas analysis system (InProcess Instruments GAM 400 mass spectrometer). Fragments with the m/z ratio of 2 (H_2_), 12 (C), 15 (CH_4_), 16 (O_2_), 18 (H_2_O), 28 (N_2_), 30 (NO), 32 (O_2_) and 44 (CO_2_) were followed. The exothermic effect of the reaction was minimized by diluting the catalyst with cordierite (150 mg, 100-150 µm).. Prior to the catalytic measurements the catalysts were degreened as follows. The mixture containing 1 vol.% CH_4_, 4 vol.% O_2_ and 95 vol.% N_2_ was fed to the catalyst at 200 ^o^C and then it was heated up to 550 ^o^C at the rate of 10^o^/min and held in these conditions for 30 min. After holding time the catalyst was cooled to the desired temperature and steam was introduced to the feed. Methane conversion curves were taken at the heating/cooling rate of 10 ^o^C per min in the feed comprising 1 vol.% CH_4_, 4 vol.% O_2_, 5 vol.% steam, N_2_ balance (GHSV = 70’000 h^-1^). Stability tests were performed in isothermal conditions (415 ^o^C) with the same feed composition. Kinetic measurements were performed within the 340-390^o^C range in the presence of water vapor in the feed and the space velocity was adjusted so that methane conversion did not exceed 10%. TOFs were calculated per atom of surface palladium which was derived from particle size distribution obtained from STEM.

**Supplementary discussion**

**X-ray diffraction.** Supplementary Figure 2 shows the XRD patterns of calcined and spent Pd/H-MOR, Pd/Na-MOR and Pd/H-MOR-BE. Only minor changes were detected in the zeolite reflection intensities (Table S1) and the loss of crystallinity was in the range of 10% irrespective of the zeolite form (H or Na). No reflections corresponding to palladium phases were detected in calcined Pd/H-MOR. Addition of sodium (Pd/Na-MOR) led to the appearance of a weak broad reflection of PdO (2θ = 33.8^o^). Whether the removal of sodium by back-exchange (Pd/H-MOR-BE) affected the dispersion of palladium is not clear from the XRD pattern, but measurable redispersion was detected by microscopy (Figure 2 and Supplementary Figure 7) and palladium K-edge EXAFS (Supplementary Figure 8). The spent materials in the acidic form (Pd/H-MOR, Pd/H-MOR-BE) exhibited sharp reflections of palladium oxide (33.8^o^) and metallic palladium (40.2^o^). In contrast, Pd/Na-MOR exhibited the same weak broad PdO reflection at 33.8^o^ as in the corresponding calcined catalyst.

**Argon physisorption.** Supplementary Figure 3 shows the argon physisorption isotherms at 77 K and the corresponding pore size distributions obtained from fitting the adsorption branches of the isotherms using the non-linear density functional theory (NLDFT) software package supplied with the instrument. Both the pore size distribution and the BET surface area (Supplementary Table 2) did not change after the 16 h stability test.

**Solid-state ^27Al^ MAS NMR.** Supplementary Figure 4 shows the ^27^Al MAS NMR spectra of calcined Pd/H-MOR, Pd/Na-MOR and Pd/H-MOR-BE and those after 16 h on stream in 1 vol. % CH_4_, 4 vol. % O_2_, 5 vol. % H_2_O, bal. N_2_ at 415 ^o^C (denoted as spent). Two distinct resonances were observed at chemical shifts of around 0 and 55 ppm. The former signal corresponds to the framework-associated octahedral aluminum (Al_oct_) and the latter to the tetrahedral framework aluminum (Al_T_). The intensity of the Al_oct_ resonance can be used to qualitatively assess the acidity of the zeolite. After exchange with sodium (Pd/Na-MOR), the intensity of Al_oct_ resonance was negligible but was restored after back-exchange (Pd/H-MOR-BE). The Al_T_ resonance slightly increased after the addition of sodium, suggesting the reinsertion of some aluminum in the framework, and after back-exchange decreased to a slightly lower level than that of the initial material. Both spent Pd/H-MOR and Pd/H-MOR-BE exhibited broadening and shift to lower values of the Al_T_ resonance as a result of the enhanced disorder in the framework aluminum. On the contrary, no difference was observed in the spectra of Pd/Na-MOR, confirming stability of the framework aluminum under reaction conditions.

**Ammonia TPD/IR.** Supplementary Figure 5 shows temperature programmed desorption curves of ammonia (NH_3_-TPD) of Pd/H-MOR, Pd/Na-MOR and Pd/H-MOR-BE. Both Pd/H‑MOR and Pd/H-MOR-BE show very similar curves with two major peaks, one in the low temperature region (weak acid sites, 100-250 ^o^C) and one in the high temperature region (strong acid sites, 250-550 ^o^C). Pd/Na-MOR exhibited predominantly a peak in the low temperature region. Addition of sodium caused almost the complete removal of the strong acid sites of the zeolite, while back-exchange (Pd/H-MOR-BE) restored the acidity profile of the initial material (Pd/H-MOR). Additionally, Pd/Na-MOR exhibited significantly larger amount of weak acid sites compared to the catalysts in the H-form.

Supplementary Figure 6 shows the OH region of the infrared spectra obtained after ammonia adsorption on Pd/H-MOR, Pd/Na-MOR and Pd/H‑MOR‑BE. The bands at 3652 and 3605 cm^-1^ (shifted to 3663 and 3615 cm^-1^ in Pd/Na-MOR) can be assigned to the bridging hydroxyl groups of the zeolite.^49,50^ The extent of titration of these groups by ammonia is significantly lower in Pd/Na-MOR than in Pd/H-MOR and Pd/H-MOR-BE confirming the presence of a lower amount of strong acid sites in the sodium form of the zeolite. The bands at 3516, 3688, 3726 and 3740 cm^-1^ correspond to the bridging OH hydroxyl groups interacting with adjacent oxygen, hydroxyl groups attached to the framework-associated aluminum, defect hydroxyl groups (“nests”) and terminal silanol groups, respectively.^49,50^ No significant difference in absorbance between H- and Na- forms of mordenite was observed for these bands.

**Palladium particle size distribution.** Supplementary Figure 7 shows the statistics of palladium particle size obtained from STEM images (Figure 2 in the main text). The initial material (Pd/H-MOR) exhibited a very narrow particle size distribution with a mean diameter of 1.3 nm. Addition of sodium (Pd/Na-MOR) caused an increase of the mean diameter to 3.0 nm. Back-exchange of sodium (Pd/H-MOR-BE) resulted in partial redispersion of palladium. Spent Pd/H-MOR exhibited a highly inhomogeneous particle size distribution with the particle size varying from 3 to 50 nm. Similarly, the spent Pd/H-MOR-BE catalyst exhibited a broad distribution of palladium particles with a mean diameter of 11 nm. In contrast, sintering was very limited in Pd/Na-MOR and a mean diameter of 4.4 and 4.6 nm after 16 and 65 h on stream, respectively, was calculated. Fresh reference Pd/Al_2_O_3_ catalyst exhibited the mean palladium particle size of 1.1 nm, whereas after 16 h this value increased to 9.2 nm.

**X-Ray absorption spectroscopy.** Supplementary Figure 8 shows the Fourier transforms of ex situ Pd K-edge EXAFS spectra. Only the first Pd–O coordination shell (ca. 1.5 Å, non-phase shift corrected) was present in Pd/H-MOR. Addition of sodium (Pd/Na-MOR) caused the appearance of the Pd–O–Pd shell (ca. 3.1 Å, non-phase shift corrected), indicating that aggregation of palladium particles occurred, whereas the back-exchange (Pd/H-MOR-BE) resulted in a decrease of the contribution of the Pd–O–Pd shell in the Fourier transform, confirming the partial redispersion. The data confirm and complement the observations and conclusions made from STEM (Figure 2 and Supplementary Figure 7) and XRD (Supplementary Figure 2).


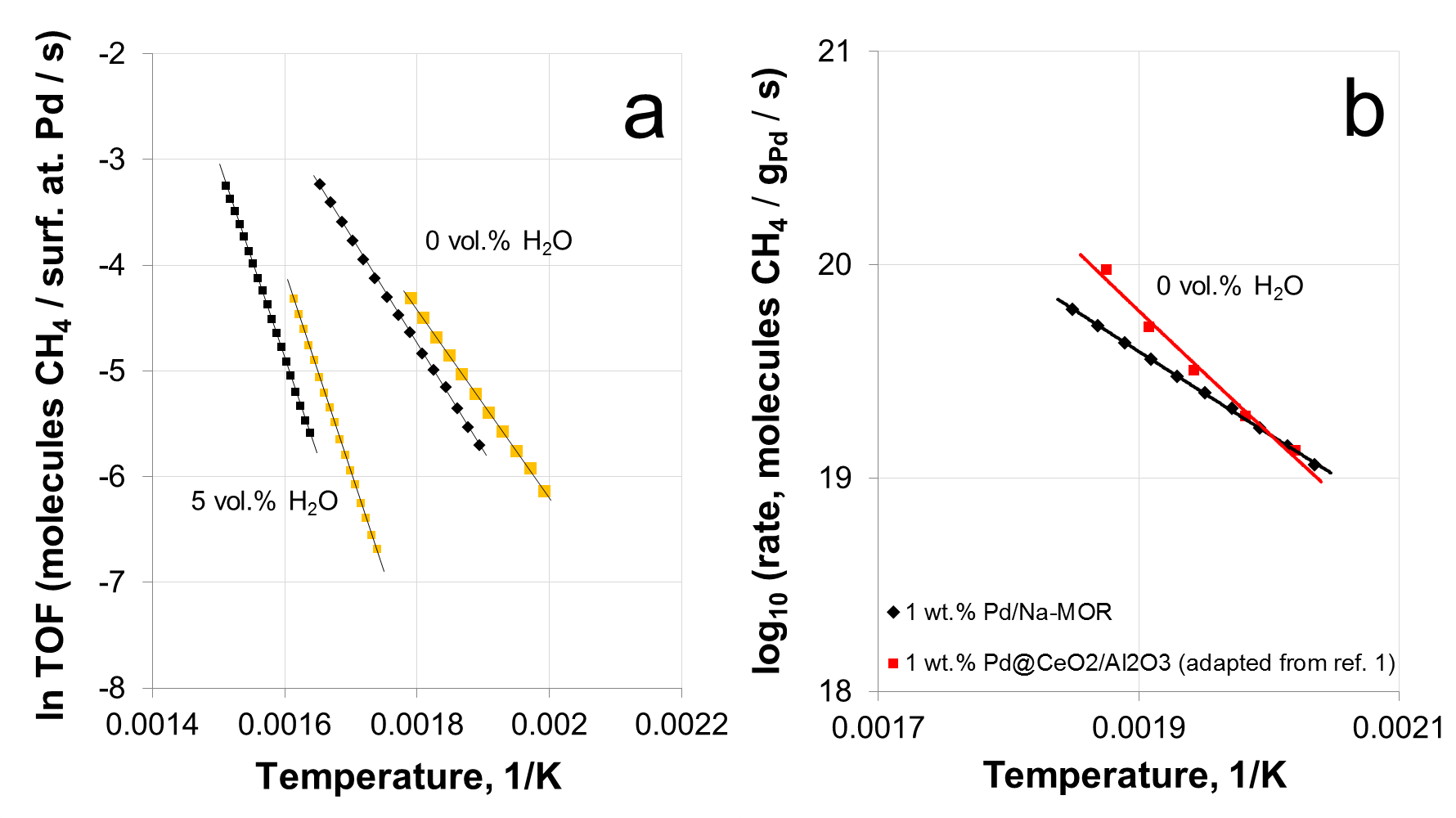


**Supplementary Figure 1.** (a) Arrhenius plots for Pd/H-MOR and Pd/Na-MOR, (b) reaction rate compared to reference 1 of the main text.


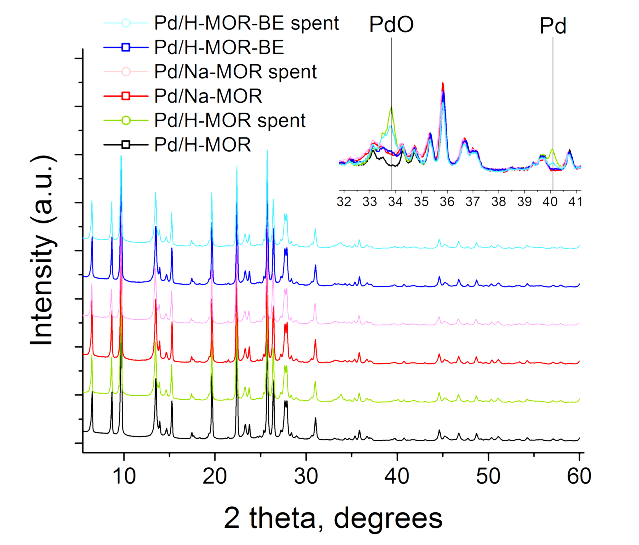


Supplementary Figure 2. XRD patterns of the calcined and spent catalysts: Pd/H-MOR, Pd/Na-MOR and Pd/H-MOR-BE. Spent catalysts have been aged in 1 vol.% CH_4_, 4 vol.% O_2_, 5 vol.% H_2_O, N_2_ bal. at 415 ^o^C for 16 h.


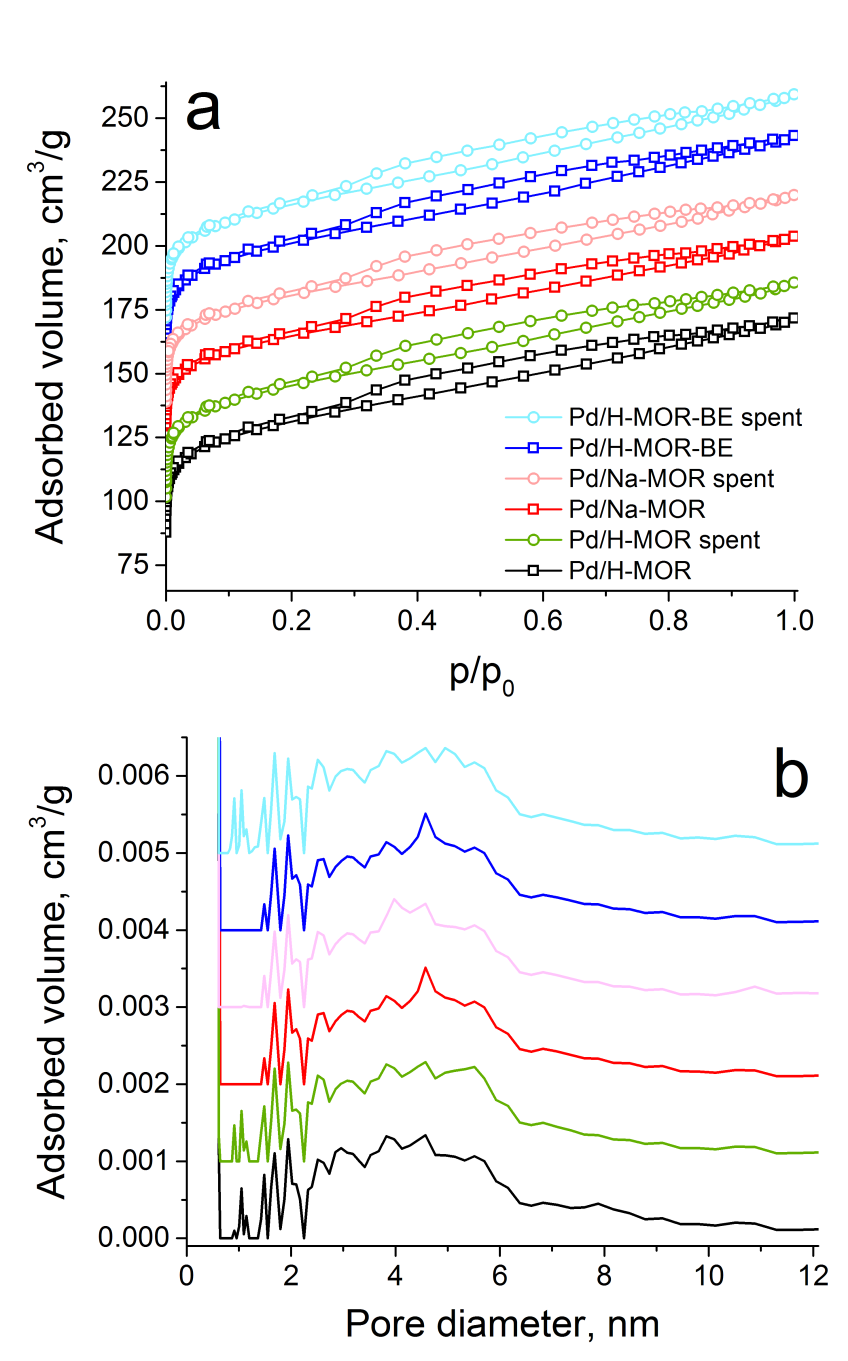


Supplementary Figure 3. (a) Argon physisorption isotherms at 77 K and (b) the corresponding pore size distributions of calcined and spent catalysts: Pd/H-MOR, Pd/Na-MOR and Pd/‑MOR‑BE. For clarity, the isotherms and the pore size distributions are offset by 25 cm^3^/g and 0.001 cm^3^/g, respectively. Spent catalysts have been aged in 1 vol.% CH_4_, 4 vol.% O_2_, 5 vol.% H_2_O, N_2_ bal. at 415 ^o^C for 16 h.


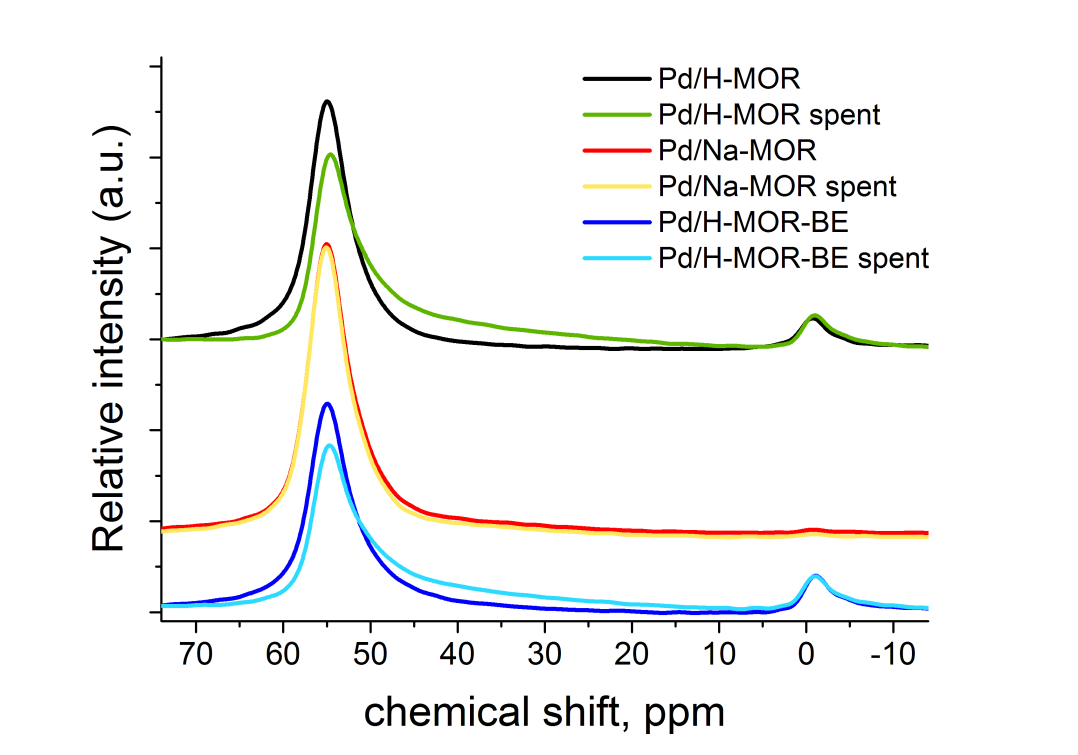


Supplementary Figure 4. ^27^Al MAS NMR spectra of calcined and spent catalysts: Pd/H-MOR, Pd/Na‑MOR and Pd/H-MOR-BE. Spent catalysts have been aged in 1 vol.% CH_4_, 4 vol.% O_2_, 5 vol.% H_2_O, N_2_ bal. at 415 ^o^C for 16 h.

**
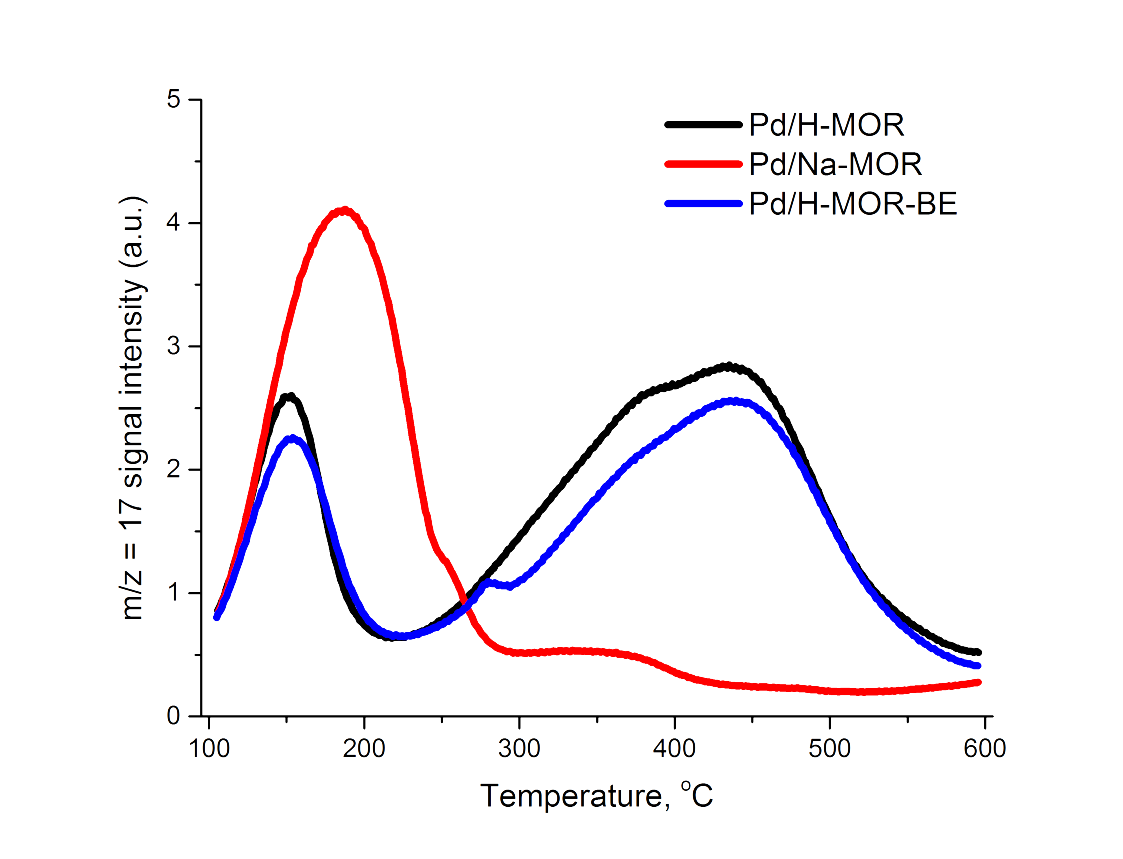
**

Supplementary Figure 5. Ammonia temperature programmed desorption thermograms (NH_3_-TPD) of Pd/H-MOR, Pd/Na-MOR and Pd/H-MOR-BE after dehydration at 550 ^o^C for 30 min and saturation with 2500 ppm of NH_3_ in nitrogen at 100 ^o^C for 15 min.


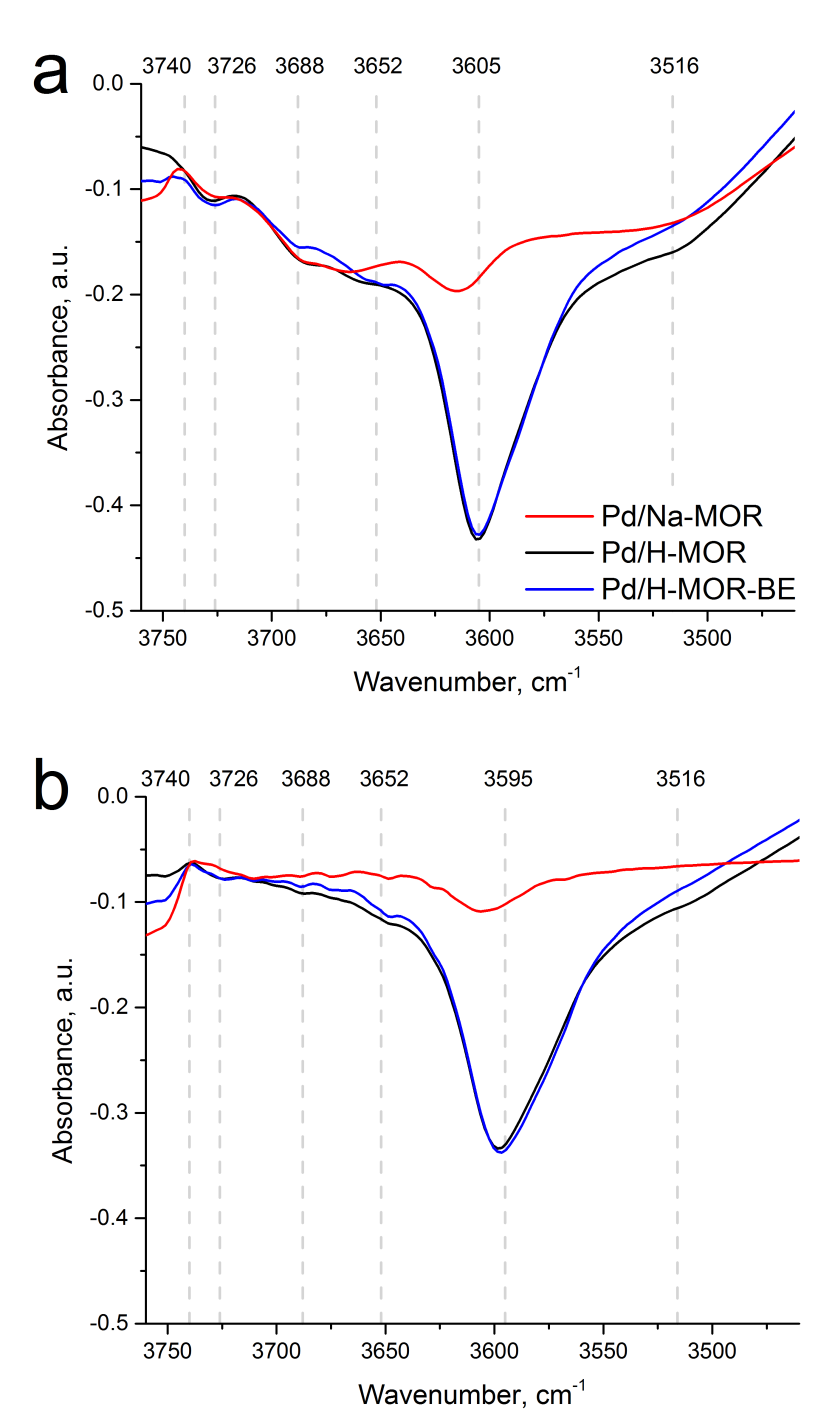


Supplementary Figure 6. Infrared spectra obtained after adsorption of ammonia on Pd/H-MOR, Pd/Na-MOR and Pd/H-MOR-BE catalysts in the 3450–3750 cm^−1^ spectral region: (a) comparison after 15 min of NH_3_ adsorption at 100 ^o^C and (b) after NH_3_ desorption in N_2_ at 300 ^o^C.

**
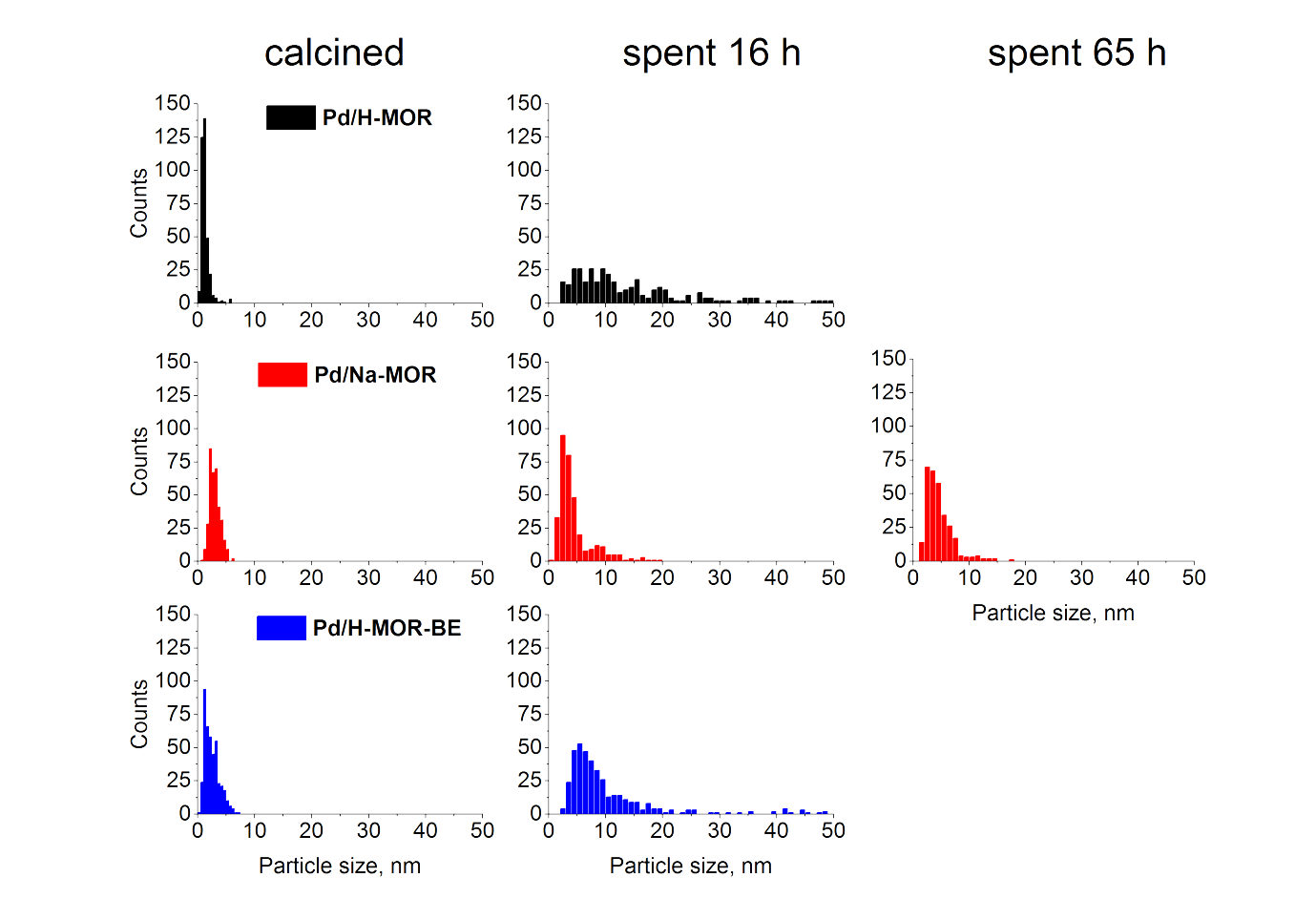
**

Supplementary Figure 7. Palladium particle size distribution in calcined and spent catalysts.


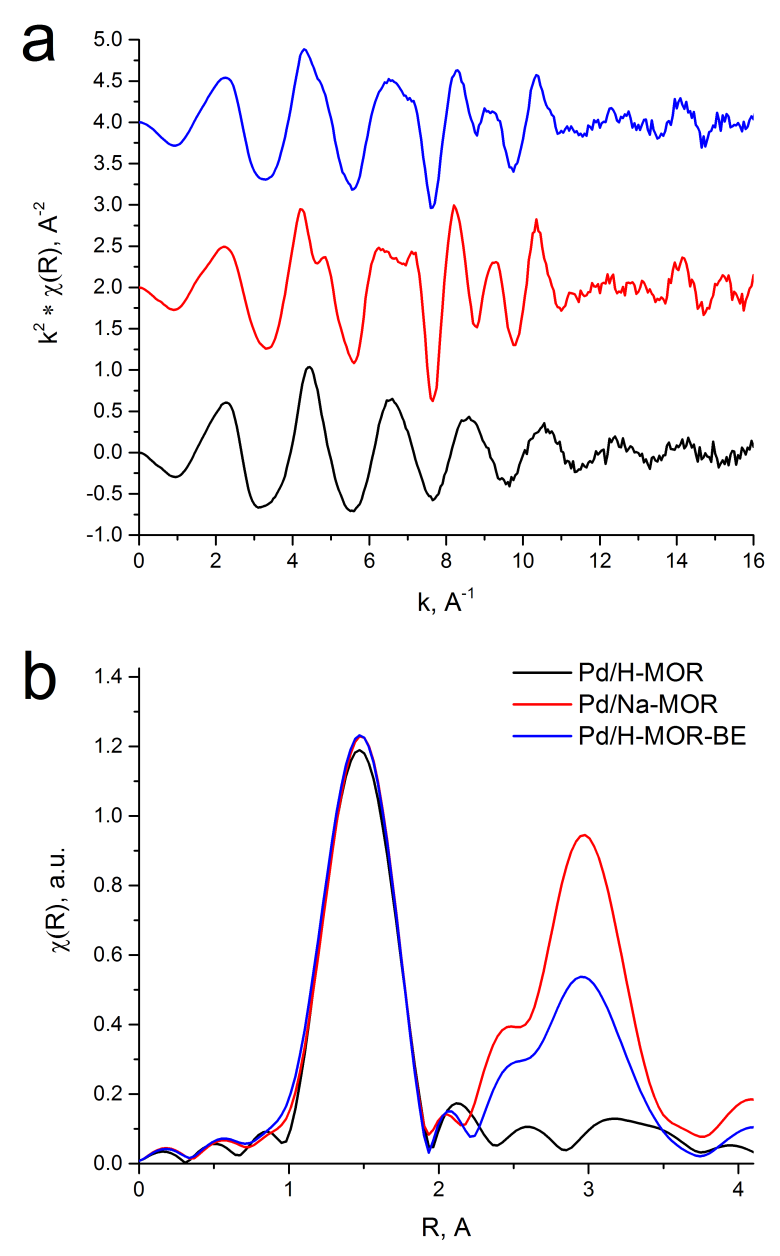


Supplementary Figure 8. Ex situ Pd K-edge EXAFS spectra of Pd/H-MOR, Pd/Na-MOR and Pd/H-MOR-BE plotted in (a) k-space and (b) R-space.


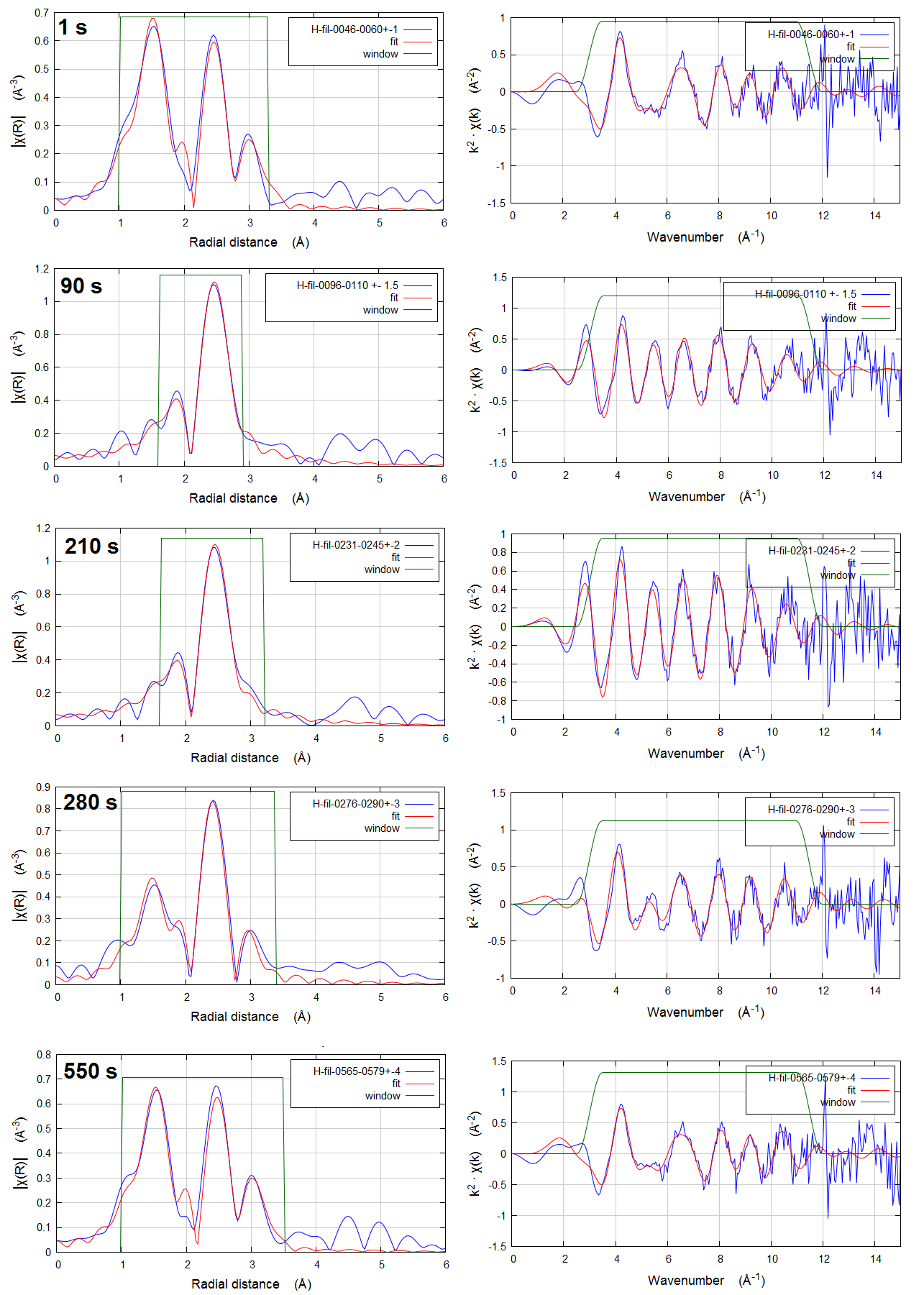


Supplementary Figure 9. Fitting of the Pd K-edge EXAFS spectra of Pd/H-MOR (at points 1, 90, 210, 280 and 550 s shown in Figure 4) in R- and k‑space.


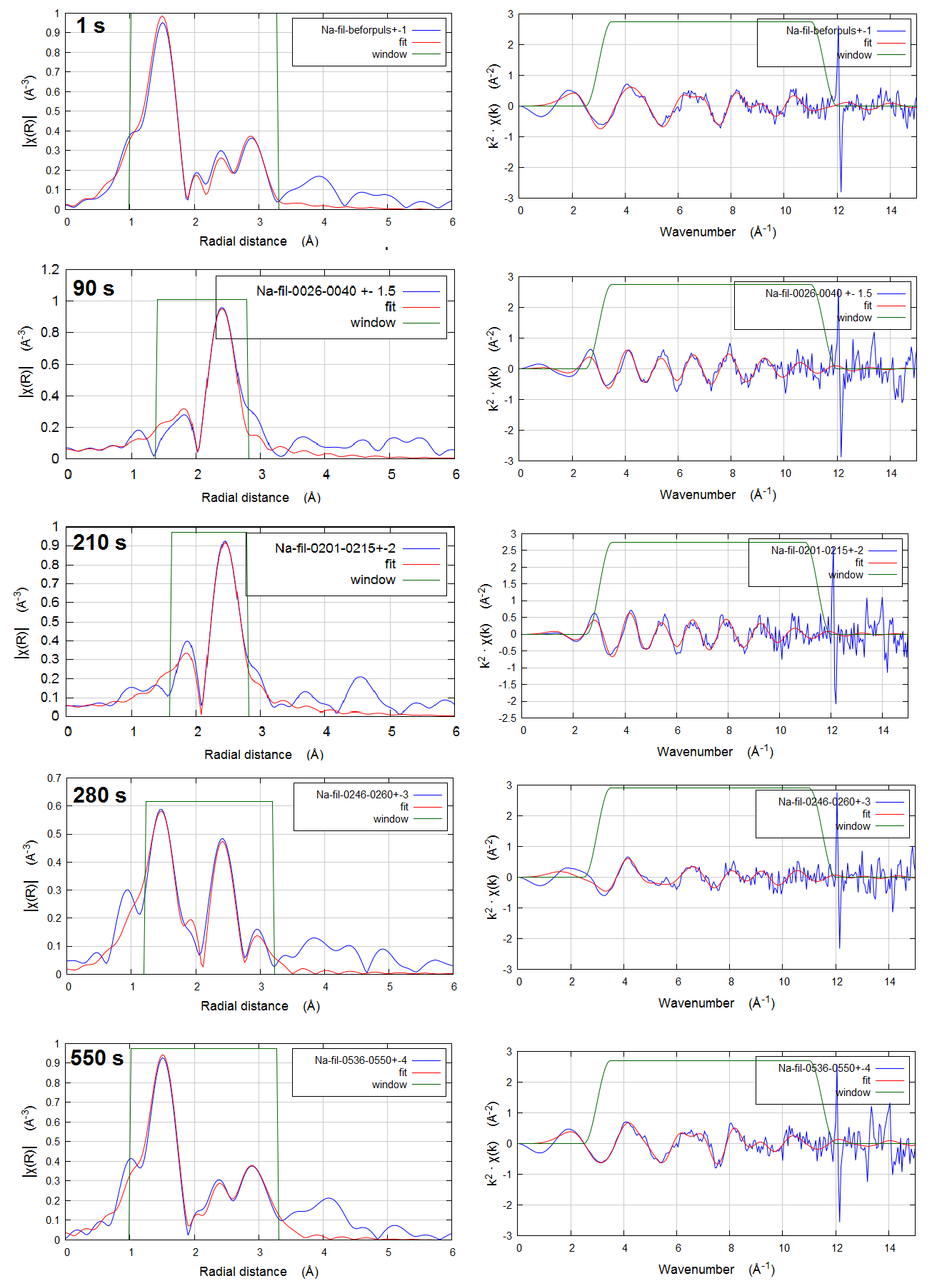


Supplementary Figure 10. Fitting of the Pd K-edge EXAFS spectra of Pd/Na-MOR (at points 1, 90, 210, 280 and 550 s shown in Figure 4) in R- and k-space.







210 s

1 s




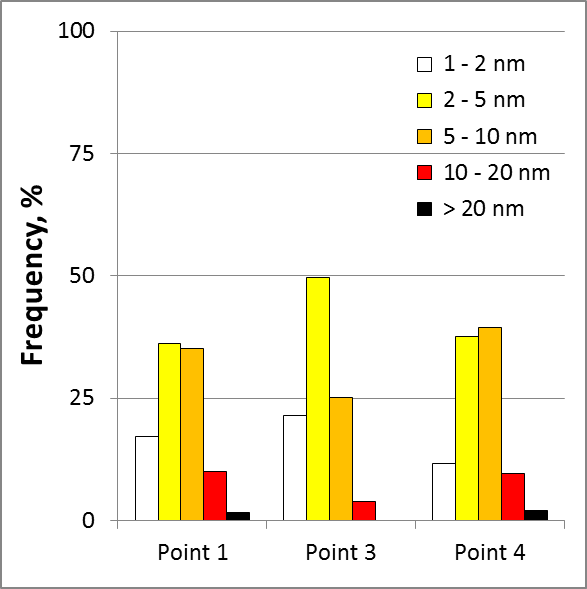


280 s

Supplementary Figure 11. Magnified STEM images from Figure 4e-g and the corresponding particle size distribution.


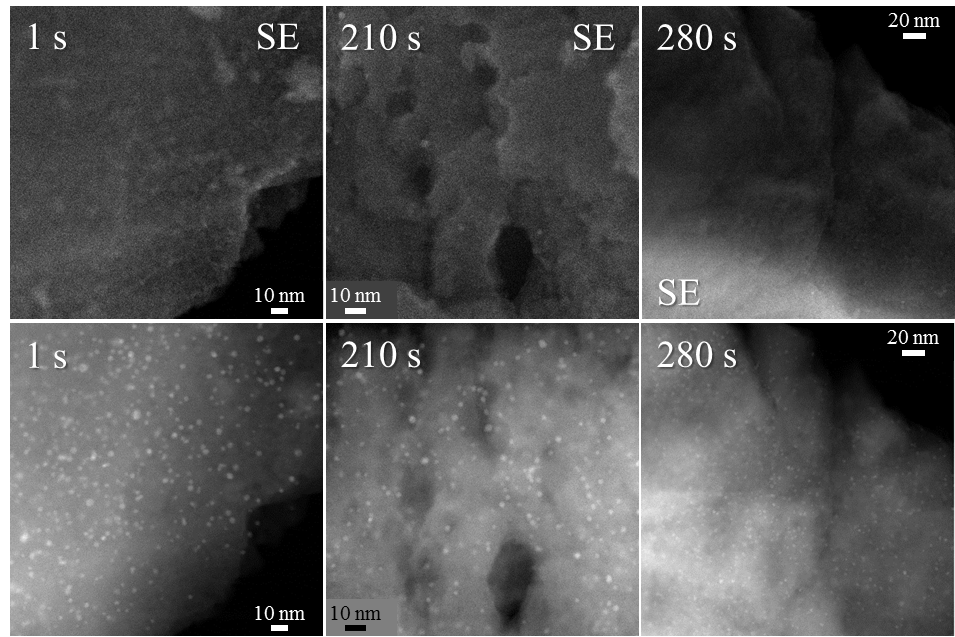


**Supplementary Figure 12.** STEM images of the quenched Pd/Na-MOR at 1, 210 and 280 s of the in situ experiment (Figure 4 of the main text). The images in top row are taken with the secondary electron detector.


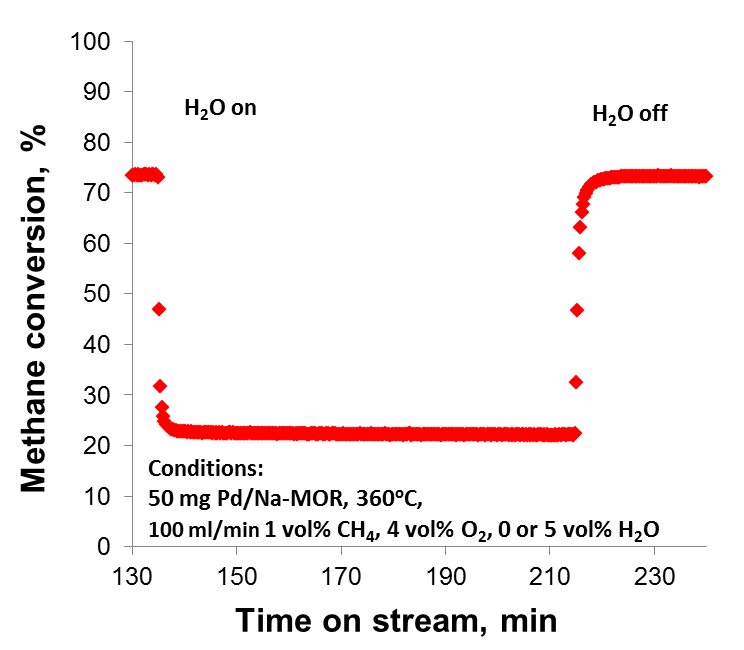


**Supplementary Figure 13.** Fully reversible effect of addition and removal of water to the feed on the activity of Pd/Na-MOR. After addition and removal of water, the activity of Pd-based catalysts is known to restore only partly due to irreversible water poisoning.^15^ In contrast, Pd/Na-MOR does not suffer from steam-induced deactivation and only exhibits reversible inhibition by water, typical for all Pd based catalysts.


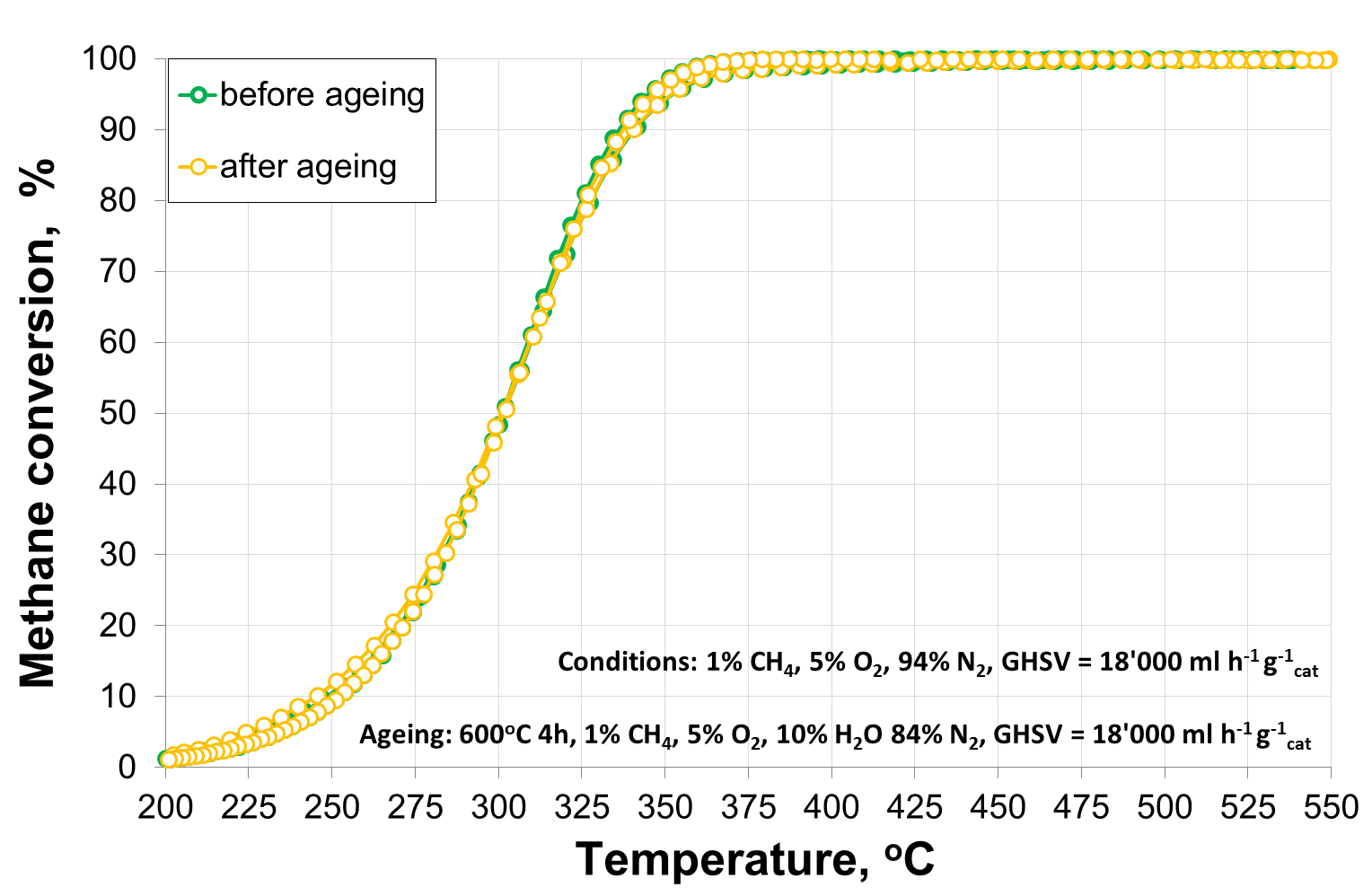


**Supplementary Figure 14.** Effect of hydrothermal ageing (600^o^C, 4h) on the activity of Pd/Na-MOR. Besides the excellent low-temperature stability, Pd/Na-MOR catalyst is also resistant to high-temperature ageing. No activity hysteresis was observed between heating and cooling curves.


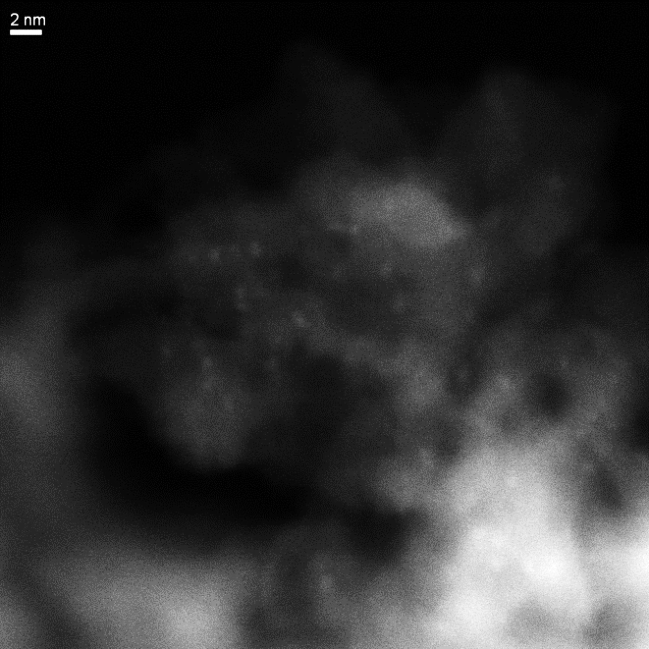









**Supplementary Figure 15.** STEM images of Pd/Al_2_O_3_ catalyst before (left) and after 16 h on stream at 415^o^C (right). The initial material exhibits nanoparticles of less than 1 nm, whereas after ageing palladium is mostly represented by ca. 5-10 nm particles. The change in activity is shown in Figure 1b of the main text.

Supplementary Table 1. Crystallinity loss of Pd/H-MOR, Pd/Na-MOR and Pd/H-MOR-BE after 16 h on stream based on the analysis of the zeolite reflection at 2θ = 22.4^o^

| **Sample** | **Calcined** | | **after 16 h on stream** | | **Crystallinity loss, %** | |
| --- | --- | --- | --- | --- | --- | --- |
|  | **Peak area** | **FWHM** | **Peak area** | **FWHM** | **From area** | **From FWHM** |
| Pd/H-MOR | 17.17 | 0.146 | 15.97 | 0.160 | 7 | 9 |
| Pd/Na-MOR | 16.45 | 0.162 | 15.28 | 0.183 | 7 | 11 |
| Pd/H-MOR-BE | 16.23 | 0.144 | 14.77 | 0.156 | 9 | 8 |

Supplementary Table 2. BET surface area of calcined and spent Pd/MOR-H, Pd/MOR-Na and Pd/MOR-H-BE from argon physisorption isotherms at 77 K

| **Sample** | **BET surface area, m^2^ g^-1^** | | **Loss of surface area, %** |
| --- | --- | --- | --- |
|  | **calcined** | **after 16 h on stream** |  |
| Pd/H-MOR | 418 | 406 | 3.0 |
| Pd/Na-MOR | 400 | 394 | 1.5 |
| Pd/H-MOR-BE | 416 | 406 | 2.5 |

Supplementary Table 3. Acidity of Pd/H-MOR, Pd/Na-MOR and Pd/H-MOR-BE calculated from NH_3_-TPD

| **Sample** | **Weak acid sites^a^, mmol/g** | **Strong acid sites^a^, mmol/g** | **Total acidity,**  **mmol/g** |
| --- | --- | --- | --- |
| Pd/H-MOR | 0.18 | 0.62 | 0.80 |
| Pd/Na-MOR | 0.42 | 0.10 | 0.52 |
| Pd/H-MOR-BE | 0.16 | 0.54 | 0.70 |

^a^ Weak and strong acid sites refer to the desorption peaks at 100-250 ^o^C and 250-550 ^o^C in the NH_3_-TPD curves, respectively (Supplementary Figure 5)

Supplementary Table 4. Chemical composition and activity of variously prepared Pd/Na-MOR catalysts

| **Sample** | **Synthesis method** | **Na content, wt. %** | **Pd content, wt. %** | **Methane conversion^a^, %** |
| --- | --- | --- | --- | --- |
| Pd/Na-MOR-EE | Na post-exchange, pH = 8.5 (excess) | 2.0 | 0.74 | < 1 |
| Pd/Na-MOR | Na post-exchange, pH = 7 | 1.4 | 0.99 | 87 |
| Pd/NaH-MOR-2 | Na pre-exchange (conventional route) | 0.8 | 0.96 | 58 |
| Pd/NaH-MOR-1 |  | 0.4 | 1.0 | 29 |
| Pd/H-MOR | – | 0.1 | 1.0 | 21 |

^a^ methane conversion after stabilization for 4 hours in the feed gas comprising 1 vol.% CH_4_, 4 vol.% O_2_, 5 vol.% H_2_O, N_2_ bal., 415^o^C

Supplementary Table 5. Fitting analysis of Pd K-edge EXAFS data

| **Material** | **Experiment** | **Comment** | **Shell** | **E_0_, eV** | **R-factor** | **CN** | **σ^2^ x 10^5^** | **ΔR** |
| --- | --- | --- | --- | --- | --- | --- | --- | --- |
| PdO | Ex situ, RT |  | Pd-O | 1.75 | 0.002 | 4 | 168 | 0.004 |
|  |  |  | Pd(-O-)Pd1 |  |  | 4 | 766 | 0.035 |
|  |  |  | Pd(-O-)Pd2 |  |  | 8 | 532 | 0.021 |
| Pd foil | Ex situ, RT |  | Pd-Pd | -5.71 | 0.001 | 12 | 517 | -0.019 |
| H-MOR | Ex situ, RT |  | Pd-O | 1.82 | 0.009 | 4 | 150 | -0.009 |
| Na-MOR | Ex situ, RT |  | Pd-O | 1.40 | 0.005 | 4 | 151 | 0.003 |
|  |  |  | Pd(-O-)Pd1 |  |  | 2 | 579 | 0.009 |
|  |  |  | Pd(-O-)Pd2 |  |  | 4 | 324 | 0.019 |
| H-MOR-BE | Ex situ, RT |  | Pd-O | 1.69 | 0.007 | 4 | 170 | -0.004 |
|  |  |  | Pd(-O-)Pd1 |  |  | 1.1 | 595 | 0.019 |
|  |  |  | Pd(-O-)Pd2 |  |  | 2.2 | 326 | 0.019 |
| Na-MOR | Ageing at 400 ^o^C (Figure 3) | 0 min | Pd-O | -1.64 | 0.009 | 3.9 | 438 | 0.000 |
|  |  |  | Pd(-O-)Pd1 |  |  | 2 | 1310 | 0.007 |
|  |  |  | Pd(-O-)Pd2 |  |  | 4 | 1284 | 0.020 |
|  |  | 90 min | Pd-O | -0.87 | 0.009 | 3.9 | 421 | 0.000 |
|  |  |  | Pd(-O-)Pd1 |  |  | 2.2 | 1338 | 0.026 |
|  |  |  | Pd(-O-)Pd2 |  |  | 4.4 | 1127 | 0.011 |
| Na-MOR | RedOx at 350 ^o^C (Figure 4) | 1 s | Pd-O | -3.39 | 0.017 | 3.9 | 337 | 0.000 |
|  |  |  | Pd(-O-)Pd1 |  |  | 2.2 | 1141 | 0.038 |
|  |  |  | Pd(-O-)Pd2 |  |  | 4.4 | 1421 | 0.003 |
|  |  | 90 s | Pd-Pd | -13.01 | 0.015 | 10.2 | 1290 | -0.053 |
|  |  | 210 s | Pd-Pd | -8.80 | 0.025 | 10.4 | 1360 | -0.039 |
|  |  | 280 s | Pd-O | -8.19 | 0.029 | 2.4 | 515 | -0.033 |
|  |  |  | Pd-Pd |  |  | 3.6 | 1058 | -0.029 |
|  |  |  | Pd(-O-)Pd1 |  |  | 0.8 | 1170 | -0.048 |
|  |  |  | Pd(-O-)Pd2 |  |  | 1.5 | 1165 | -0.005 |
|  |  | 550 s | Pd-O | -3.01 | 0.011 | 3.6 | 330 | 0.005 |
|  |  |  | Pd-Pd |  |  | 1.2 | 1136 | -0.044 |
|  |  |  | Pd(-O-)Pd1 |  |  | 2.3 | 1288 | 0.012 |
|  |  |  | Pd(-O-)Pd2 |  |  | 4.6 | 1216 | 0.033 |
| H-MOR | Ageing at 400 ^o^C (Figure 3) | 0 min | Pd-O | -4.99 | 0.025 | 2.9 | 460 | -0.004 |
|  |  |  | Pd-Pd |  |  | 0.5 | 1150 | 0.010 |
|  |  |  | Pd(-O-)Pd1 |  |  | 0.3 | 1300 | -0.024 |
|  |  |  | Pd(-O-)Pd2 |  |  | 0.7 | 1270 | -0.078 |
|  |  | 15 min | Pd-O | -4.90 | 0.017 | 2.9 | 474 | -0.004 |
|  |  |  | Pd-Pd |  |  | 1.6 | 1150 | -0.007 |
|  |  |  | Pd(-O-)Pd1 |  |  | 0.9 | 1300 | -0.002 |
|  |  |  | Pd(-O-)Pd2 |  |  | 1.8 | 1270 | 0.010 |
|  |  | 30 min | Pd-O | -2.42 | 0.025 | 2.9 | 463 | 0.007 |
|  |  |  | Pd-Pd |  |  | 1.9 | 1150 | 0.032 |
|  |  |  | Pd(-O-)Pd1 |  |  | 1.4 | 1300 | 0.015 |
|  |  |  | Pd(-O-)Pd2 |  |  | 2.8 | 1270 | 0.012 |
|  |  | 90 min | Pd-O | -2.32 | 0.037 | 3.0 | 447 | 0.010 |
|  |  |  | Pd-Pd |  |  | 2.8 | 1150 | 0.019 |
|  |  |  | Pd(-O-)Pd1 |  |  | 2.3 | 1300 | 0.004 |
|  |  |  | Pd(-O-)Pd2 |  |  | 4.6 | 1271 | 0.026 |
| Continued on the next page | | | | | | | | |
| Supplementary Table 5 (continued) | | | | | | | | |
| H-MOR | RedOx at 350 ^o^C (Figure 4) | 1 s | Pd-O | -3.66 | 0.039 | 2.4 | 380 | 0.000 |
|  |  |  | Pd-Pd |  |  | 4.6 | 1027 | 0.001 |
|  |  |  | Pd(-O-)Pd1 |  |  | 1.8 | 1250 | -0.021 |
|  |  |  | Pd(-O-)Pd2 |  |  | 3.5 | 1350 | 0.015 |
|  |  | 90 s | Pd-Pd | -7.97 | 0.007 | 11.3 | 1260 | -0.033 |
|  |  | 210 s | Pd-Pd | -8.84 | 0.013 | 11.3 | 1270 | -0.038 |
|  |  | 280 s | Pd-O | -9.50 | 0.040 | 1.4 | 380 | -0.020 |
|  |  |  | Pd-Pd |  |  | 7.0 | 1030 | -0.030 |
|  |  |  | Pd(-O-)Pd1 |  |  | 1.3 | 1250 | -0.071 |
|  |  |  | Pd(-O-)Pd2 |  |  | 2.6 | 1350 | 0.038 |
|  |  | 550 s | Pd-O | -2.81 | 0.042 | 2.3 | 380 | 0.005 |
|  |  |  | Pd-Pd |  |  | 4.9 | 1030 | 0.015 |
|  |  |  | Pd(-O-)Pd1 |  |  | 2.1 | 1250 | -0.006 |
|  |  |  | Pd(-O-)Pd2 |  |  | 4.2 | 1350 | 0.012 |

Supplementary Table 6. Analysis of STEM images from Figure 4e-g

| Time in Figure 4 | Mean palladium particle diameter, nm | Round form (%) | Irregular form (%) |
| --- | --- | --- | --- |
| 1 s | 6.4 | 65 (29%) | 161 (71%) |
| 210 s | 4.9 | 195 (67%) | 96 (33%) |
| 280 s | 6.7 | 77 (40%) | 115 (60%) |

**Supplementary references**

1 Ravel, B. & Newville, M. ATHENA, ARTEMIS, HEPHAESTUS: data analysis for X-ray absorption spectroscopy using IFEFFIT. *J. Synchrotron Rad.* **12**, 537-541 (2005).

2 Sandoval-Díaz, L.-E., González-Amaya, J.-A. & Trujillo, C.-A. General aspects of zeolite acidity characterization. *Micropor. Mesopor. Mat.* **215**, 229-243 (2015).

3 Karge, H. G. in *Verified Syntheses of Zeolitic Materials* 69-71 (Elsevier Science, 2001).
