# Supplementary material for: Stable complete methane oxidation over palladium based zeolite catalysts
Source: Nat Commun. 2018 Jun 29;9:2545. doi: 10.1038/s41467-018-04748-x (PMC6026177; doi:10.1038/s41467-018-04748-x)
Supplement: Supplementary file 2 — Peer Review File [file 41467_2018_4748_MOESM2_ESM.pdf]

Editorial Note: This manuscript has been previously reviewed at another journal that is not operating a transparent peer review scheme. This document only contains reviewer comments and rebuttal letters for versions considered at Nature Communications. Mentions of prior referee reports have been redacted.

PEER REVIEW FILE

Reviewers' Comments:

Reviewer #1 (Remarks to the Author):

The authors have presented a satisfactory rebuttal that addresses all relevant technical questions. Whether this technology actually makes it to commercial application is a separate matter that is not relevant for a scientific journal. Many other factors will enter into consideration. As far as I can see, the work represents a technical advance and presents a strategy to combat deactivation induced under reaction conditions. I find the manuscript suitable for publication.
